# Appendix B* Some initial recommendations for potential Women's Health and Disease Surveillance Indicators

**DOI:** 10.1186/1472-6874-4-S1-S36

**Published:** 2004-08-25

**Authors:** Marie Desmeules

**Affiliations:** 1 Centre for Chronic Disease Prevention and Control, Health Canada, 120 Colonnade Rd, Ottawa, Canada

## 

Below are some examples of possible indicators for assessing women's health in a gender relevant way.

## Health Determinants

▪ Percentage of women engaging in specific multiple risk practices (such as binge drinking, smoking and physical inactivity) by age, educational level, household income adequacy, marital status, ethnic background and region.

▪ Percentage of women engaging in specific multiple health promoting practices (such as being physically active and consuming more than five servings of fruits and vegetables per day) by age, educational level, household income adequacy, marital status, ethnic background and region.

▪ Self reported physical activity levels by age, sex, educational level, household income adequacy, ethnic background, marital status (for all ages) which includes measures of daily physical activity (e.g. household chores, school based activity, activity at work etc.).

▪ Self-reported dietary habits (such as consumption of fruits and vegetables and high fat foods) by age, sex, educational level, household income adequacy, ethnic background, marital status.

▪ Percentage of women endorsing the following reasons for physical activity: physical fitness, health, lifestyle, social, enjoyment, appearance, weight control.

▪ Percentage of women who report being satisfied with their body weight.

▪ Measured BMI values by age, sex, educational level, household income adequacy, ethnic background, marital status.

▪ Percentage of women working part-time (vs. full-time) by age, educational level, household income adequacy, ethnic background and marital status.

▪ Percentage of women with flexible work hours by age, educational level, household income adequacy, ethnic background and marital status.

▪ Percentage of women engaged in on-call or shift work by age, educational level, household income adequacy, ethnic background and marital status.

▪ Percentage of intergenerational households by age, educational level, household household income adequacy, ethnic background and marital status.

▪ Percentage of households headed by same sex couples.

▪ Percentage of households engaging in multi-family arrangements.

▪ Percentage of women satisfied with their partnership by age, educational level, household income adequacy, ethnic background and marital status.

▪ F/M Ratio of average time spent on household work by age, educational level, household income.

▪ Average Number of years of current marriage/partnership by age, educational level, household income adequacy and ethnic background.

▪ Percentage of smokers 12+ by age group, sex, ethnic background, income and education.

▪ Percentage of women who use [specific licit and illicit substances] by age, educational level, household income adequacy, ethnic background and marital status.

▪ Percentage of women who smoke during pregnancy.

▪ Percentage of women who use [specific licit and illicit substances] during pregnancy.

## Health Status

▪ Life Expectancy and Health Adjusted Life Expectancy by sex.

▪ Overall mortality and preventable mortality rates by sex and rural/urban residence.

▪ Cause-deleted life expectancy and Cause-deleted Health Adjusted Life Expectancy (by sex) – deleting "exogenous" (preventable through primary prevention) causes; "endogenous" (sex/biology related) causes, and causes amenable to health care, by sex.

▪ Percentage of births to women 35+ years, age group, region, education, household income adequacy, marital status and ethnic background.

▪ Average maternal age at 1st birth, by province/territory, education, occupation, marital status and ethnic background.

▪ Hospital separation rates for all causes except pregnancy and childbirth without complications (among women).

▪ Hospital separation rates for ambulatory-care sensitive conditions, by sex.

▪ Prevalence of disability/activity limitation, by severity and sex.

▪ Odds ratio of disability by sex, adjusted for risk factors such as chronic conditions and smoking.

▪ Prevalence of disability by household income adequacy, employment, family structure and social support among women and men.

▪ Prevalence of underlying conditions among women and men with a disability.

## Women's Health Related Conditions

▪ Self reported health status by age, ethnic background and educational level, household income adequacy, among pre, peri and post-menopausal women.

▪ Rates of fragility fractures (hip and wrist) by age, ethnicity, SES, and previous fracture among post-menopausal women, or women aged 50 or older.

▪ Survival rates (e.g. 5-year) and stage at diagnosis for breast cancer, other females cancers, and other cancers (such as lung cancer).

▪ Rates of mortality due to stroke by age, ethnic background, SES, and previous stroke.

▪ Percentage women who have probable# depression who obtain treatment for depression from a Health Care Practioner.

▪ Percentage women who have been diagnosed† with depression who obtain treatment for depression from a Health Care Practioner.

▪ Prevalence of reported anxiety disorders by age and sex.

▪ Prevalence of treated anxiety disorders by age and sex.

▪ Prevalence of depression and anxiety treatment in the year after childbirth.

▪ Number of workdays lost among those who report stress, anxiety and depression by age and sex in the last year.

▪ Percentage of victims of violence prescribed psychoactive medication post event by sex.

▪ Percentage of women with a BMI less than 25 and who perceive themselves to be overweight.

▪ Percentage of women with a BMI less than 20 who perceive themselves to be average weight.

▪ Average reported age at first penetrative intercourse by sex and ethnic group.

▪ Percentage of individuals having 2+ partners in a 12 month period (multiple partner).

▪ Percentage of individuals having 2+ partners in 12 months + inconsistent use of barrier method of contraception by sex, age & ethnic group.

▪ Percentage of individuals with multiple partners who consistently use condoms.

▪ Type of contraceptive used by women by marital status, age, ethnic background and sexual orientation.

▪ Prevalence of hypertension and lipid profile by age, ethnic background and educational level, household income adequacy;.

▪ The F/M Ratio of individuals undergoing stress tests, angiograms, echocardiography, and holter monitoring;.

▪ Prevalence of comorbid depression and anxiety, and/or treatment via psychotherapy and pharmacotherapy among women with CVD/CBVD.

▪ Prevalence of national drug use for the treatment and prevention of CVD/CBVD (and subsequent control rate for hypertension & hyperlipidemia) among women;.

▪ Incidence of long-term cardiac rehabilitation clinical outcomes in women.

▪ Chlamydia incidence rate in women between 15 and 24 years.

▪ Percentage of positive HIV test in women by age, ethnic background, possible risk factor (such as injection drug use, bisexuality, multiple partners).

▪ Prevalence of women who had an HIV test in the last year/ or ever, by age group, ethnic background and possible risk factor.

▪ Prevalence of partners who had an HIV test in the last year/ or ever, by age group, ethnic background and possible risk factor.

▪ Prevalence of risk behaviours among women and their partners (for example) injection drug use, bisexuality, multiple partners.

## General

▪ Percentage of population aged 20+ with < HS education by sex, age, province/territory, ethnic background.

▪ Percentage of population aged 20+ who is not a student and not in the workforce by sex, age, region, ethnic background.

▪ Percentage of households headed by women by types of households.

▪ Percentage of households with children under five years of age.

▪ Percentage of households with people requiring long standing care by (number and age of dependants).

▪ Percentage of households with home crowding (number and age of dependants).

▪ F/M Ratio of average earnings by parity.

▪ Percentage of individuals at specific distance categories from health centre by region, by sex.

## Note

* The views expressed in this report do not necessarily represent the views of the Canadian Population Health Initiative, the Canadian Institute for Health Information or Health Canada.

# From health surveys

† From health services databases

